# Editorial: Harnessing marine biodiversity for novel antimicrobial agents against multidrug-resistant pathogens

**DOI:** 10.3389/fmicb.2025.1607539

**Published:** 2025-04-24

**Authors:** Guillermin Agüero-Chapin, Dany Domínguez-Pérez, Yovani Marrero-Ponce

**Affiliations:** ^1^CIIMAR - Centro Interdisciplinar de Investigação Marinha e Ambiental, Universidade do Porto, Porto, Portugal; ^2^Department of Biology and Evolution of Marine Organisms (BEOM), Stazione Zoologica Anton Dohrn, Calabria Marine Centre (CRIMAC), Amendolara, Italy; ^3^PagBiOmicS - Personalised Academic Guidance and Biodiscovery-Integrated OMICs Solutions, Porto, Portugal; ^4^Universidad Panamericana, Facultad de Ingeniería, Ciudad de México, Mexico; ^5^Universidad San Francisco de Quito (USFQ), Grupo de Medicina Molecular y Traslacional (MeM&T), Colegio de Ciencias de la Salud (COCSA), Escuela de Medicina, Quito, Ecuador

**Keywords:** multidrug resistance, marine antimicrobials, bioassays, screening techniques, drug discovery

Antimicrobial resistance (AMR) is a defining challenge of our era, responsible for an alarming number of deaths that now surpass those caused by HIV and malaria. Projections estimate that by 2050, AMR could lead to 10 million deaths annually. The COVID-19 pandemic has amplified this crisis, fueling the spread of multidrug-resistant (MDR) pathogens, particularly those associated with biofilms. In response, governments have begun adopting more agile investment models, while academia and emerging biotech initiatives play increasingly central roles in the discovery of next-generation antimicrobials.

The ocean, covering over 70% of Earth's surface, represents an extraordinary yet underexploited reservoir of chemical diversity. Marine ecosystems harbor a vast array of microorganisms and multicellular life forms adapted to extreme and varied habitats. These organisms, from actinomycetes to fish and fungi, produce structurally unique secondary metabolites as chemical defenses or communication tools—many of which exhibit promising antimicrobial activities. This Research Topic aims to showcase the potential of marine biodiversity in providing new solutions to counteract MDR pathogens.

## Thematic contributions

This collection of seven peer-reviewed articles exemplifies the multidisciplinary approaches required to unlock the antimicrobial potential of the ocean.

Marine actinomycetes, long recognized as prolific producers of bioactive compounds, are at the forefront of this exploration. In their mini review, Pan et al. catalog 45 novel antibacterial compounds identified in 2024 from marine actinomycetes, such as polyketides, macrolactams, alkaloids and peptides. The review highlights the origins, chemical structures, and biological activities of these metabolites. Their distinct structural features and potent antibacterial properties, along with detailed insights into their mechanisms of action, underscore their potential as promising leads to combat antimicrobial resistance.

De La Hoz-Romo et al. extended this work through an application-focused study investigating marine actinobacteria isolated from the sponge *Cliona varians* and the octocoral *Eunicea fusca* for their activity against acne-associated bacteria, including *Cutibacterium acnes, Staphylococcus epidermidis*, and methicillin-resistant *Staphylococcus aureus* (MRSA). Notably, the extract Z9.216 from *Kocuria* sp. exhibited antibacterial activity comparable to erythromycin and vancomycin, without cytotoxic effects on human keratinocytes and fibroblasts at effective concentrations. These results underscore the therapeutic promise of rare marine actinobacteria, with alkaloids and terpenoids likely contributing to the observed bioactivity.

Similarly, Pylkkö et al. employed antivirulence screening to identify metabolites from Arctic marine actinobacteria—*Kocuria* sp. and *Rhodococcus* spp.—capable of inhibiting *Escherichia coli* (EPEC) pathogenicity without affecting bacterial growth. EPEC, a major cause of infant intestinal infections in developing countries, induces epithelial lesions through actin polymerization. Using bioassay-guided fractionation and HPLC-MS dereplication, the study identified a large phospholipid and a likely antimicrobial peptide that interfered with EPEC-induced actin remodeling. These results reinforce the potential of antivirulence approaches to limit resistance by avoiding direct effects on bacterial viability.

Expanding the chemical space further, Wang et al. reviewed 337 secondary metabolites isolated from marine-derived *Aspergillus* species between 2010 and mid-2024, including 145 new compounds. Classified into terpenoids, nitrogen-containing compounds, polyketides, steroids, and others, these metabolites display notable antibacterial activities. Their structural diversity and bioactivity highlight the valuable but underexplored role of marine fungi in antimicrobial drug discovery.

Liu et al. focused on another marine-derived fungus *Trichoderma effusum*, isolating four new sesquiterpene derivatives—trichoderenes A–D—alongside six known compounds. Several of these, including compounds 1–3 and 8–10, exhibited inhibitory activity against *Agrobacterium tumefaciens*, a phytopathogen responsible for significant agricultural losses. Notably, compound 3 introduced a previously undescribed C12 nor-sesquiterpene skeleton, underscoring the structural novelty and bioactive potential accessible from marine fungal sources.

A promising example of marine-derived antimicrobial peptides (AMPs) comes from Squitieri et al., who engineered two cationic mutants—Trem-HK and Trem-HSK—based on Trematocine, a natural AMP from the Antarctic fish *Trematomus bernacchii*. These designed peptides exhibited enhanced selectivity for bacterial membranes, preserved α-helical structure, and markedly improved efficacy against ESKAPE pathogens, with MIC and MBC values reduced by up to 80% compared to the original peptide. Notably, both mutants demonstrated low cytotoxicity and hemolytic activity at effective concentrations, and showed no *in vivo* toxicity in *Galleria mellonella* larvae, supporting their potential as promising leads for antimicrobial drug development.

Finally, He et al. demonstrated the integration of nanotechnology with marine bioproducts by synthesizing silver nanoparticles (PSP-AgNPs) from a polysaccharide-protein complex of the marine mollusk *Haliotis discus*. These nanoparticles showed strong antibacterial activity against several *Vibrio* strains, including *V. fluvialis, V. mimicus, V. hollisae, V. vulnificus*, and *V. furnissii*, with no cytotoxic effects on human hepatocytes at effective dosages (3.125–25.0 μg/mL), underscoring their potential as biocompatible bactericides for public health.

## Conclusions and future perspectives

The articles in this Topic highlight the diverse sources, strategies, and applications of marine biodiversity in antimicrobial development. While the potential is clear, challenges remain, including sustainable sourcing, low natural yields, and complex compound isolation. Overcoming these obstacles requires interdisciplinary approaches that combine omics, synthetic biology, machine learning, and cheminformatics, as well as policy support for responsible bioprospecting and resource management.

This Research Topic emphasizes that the ocean, while a vital source of life, may also hold the key to combating one of the most pressing health threats of our time. Continued investment in marine biodiscovery is not just an academic pursuit—it is critical for global public health.

